# A case of acute obstructive pyelonephritis due to bleeding from a renal pelvis cancer rescued with laparoscopic nephrectomy

**DOI:** 10.1002/iju5.12816

**Published:** 2025-05-02

**Authors:** Masato Yanagi, Ken Takahashi, Yuta Kikuchi, Takuya Takahashi, Hideaki Iwata, Hiro Izuta, Shuichi Osawa, Taiji Nishimura, Yukihiro Kondo

**Affiliations:** ^1^ Department of Urology Heisei‐Tateishi Hospital Katsushika‐ku Tokyo Japan; ^2^ Department of Urology Nippon Medical School Hospital Bunkyo‐ku Tokyo Japan; ^3^ Department of Surgery Heisei‐Tateishi Hospital Katsushika‐ku Tokyo Japan; ^4^ Department of Anesthesiology Heisei‐Tateishi Hospital Katsushika‐ku Tokyo Japan

**Keywords:** laparoscopic nephrectomy, obstructive pyelonephritis, renal pelvic tumor

## Abstract

**Introduction:**

We encountered a case of acute obstructive pyelonephritis caused by bleeding from a renal pelvis cancer that was successfully treated by laparoscopic nephrectomy.

**Case presentation:**

An 88‐year‐old woman with fever of 40.2°C and right back pain associated with hematuria due to a right renal pelvic tumor. Computed tomography showed a blood clot filling the right renal pelvis and ureter. She was diagnosed with severe obstructive pyelonephritis due to undrainable blood clots. Nephrectomy was performed to control the infection. Although the perirenal area was easy bleeding, nephrectomy was completed and the patient's condition improved.

**Conclusion:**

Renal pelvis carcinomas with hemorrhage requiring blood transfusion should be treated with radical nephroureterectomy as early as possible.

**Keynote message**

Radical nephroureterectomy should be performed as soon as possible in patients with hematuria from renal pelvic carcinoma requiring blood transfusion.

**Abbreviations and Acronyms**
CT: computed tomography
DIC: disseminated intravascular coagulation
ECOG PS: Eastern Cooperative Oncology Group performance status
WBCs: white blood cells

## Introduction

Hematuria is a common symptom of renal pelvis cancer.^1^ However, it is very rare for renal pelvis cancer to cause acute obstructive pyelonephritis due to massive bleeding. We encountered a case of acute obstructive pyelonephritis caused by bleeding from a renal pelvis cancer that was successfully treated by laparoscopic nephrectomy.

## Case presentation

**Fig. 1:**
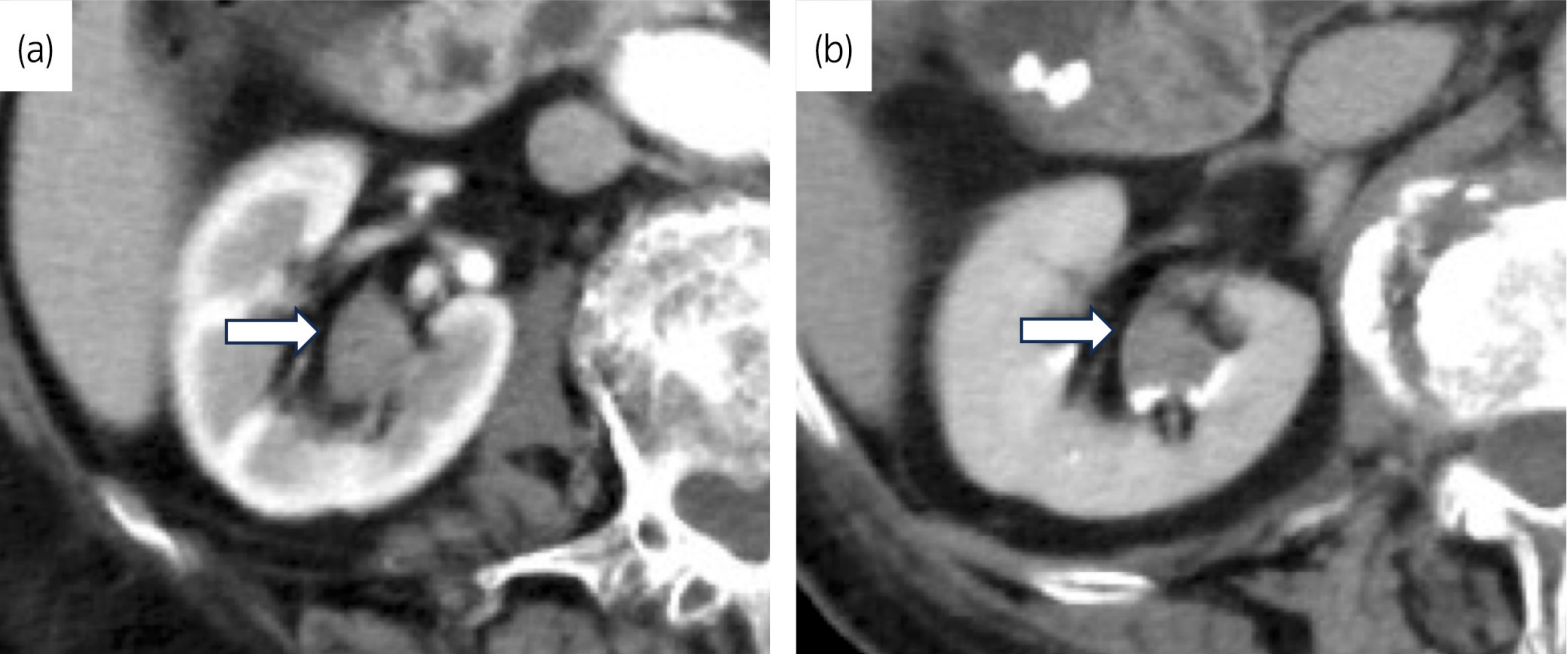
Findings of contrast‐enhanced CT at initial diagnosis of renal pelvic tumor. (a) Arterial phase of contrast enhanced CT. A white arrow shows renal pelvic tumor. (b) Excretory phase of contrast enhanced CT. A white arrow shows renal pelvic tumor.

**Fig. 2:**
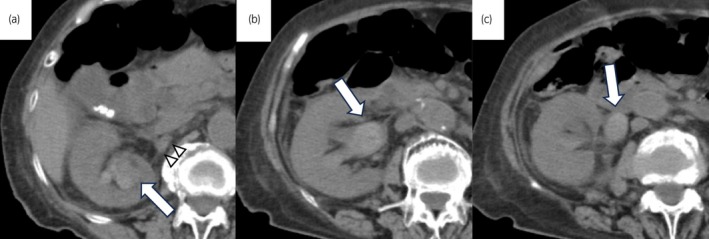
Findings of CT at acute obstructive pyelonephritis. (a) A white arrow shows a right renal pelvic tumor with active bleeding. White arrowheads show mild strandings around the renal artery. (b) A white arrow shows a dilated right renal pelvis due to blood clots. (c) A white arrow shows a dilated right ureter due to blood clots.

**Fig. 3:**
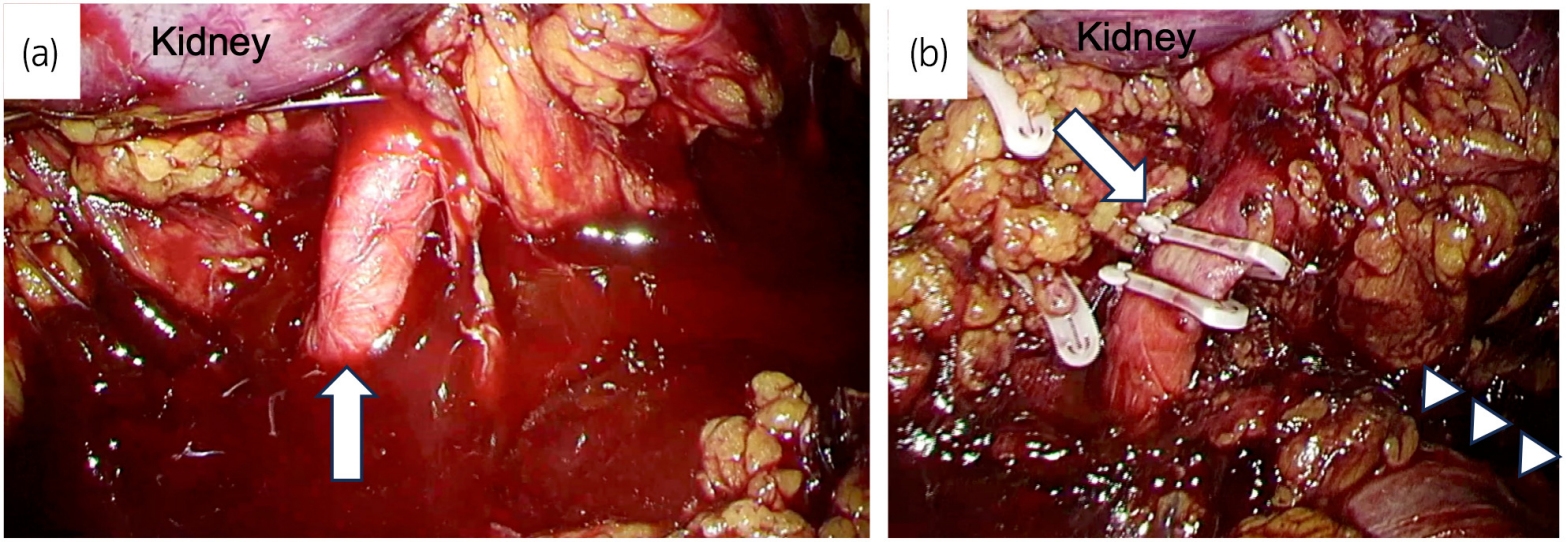
Intraoperative findings. (a) A white arrow shows a right renal artery. The area around a right renal hilum was easy to hemorrhage. (b) A white arrow shows a right renal artery clipped with XL Hem‐o‐lok clips. White arrowheads show an inferior vena cava.

**Fig. 4:**
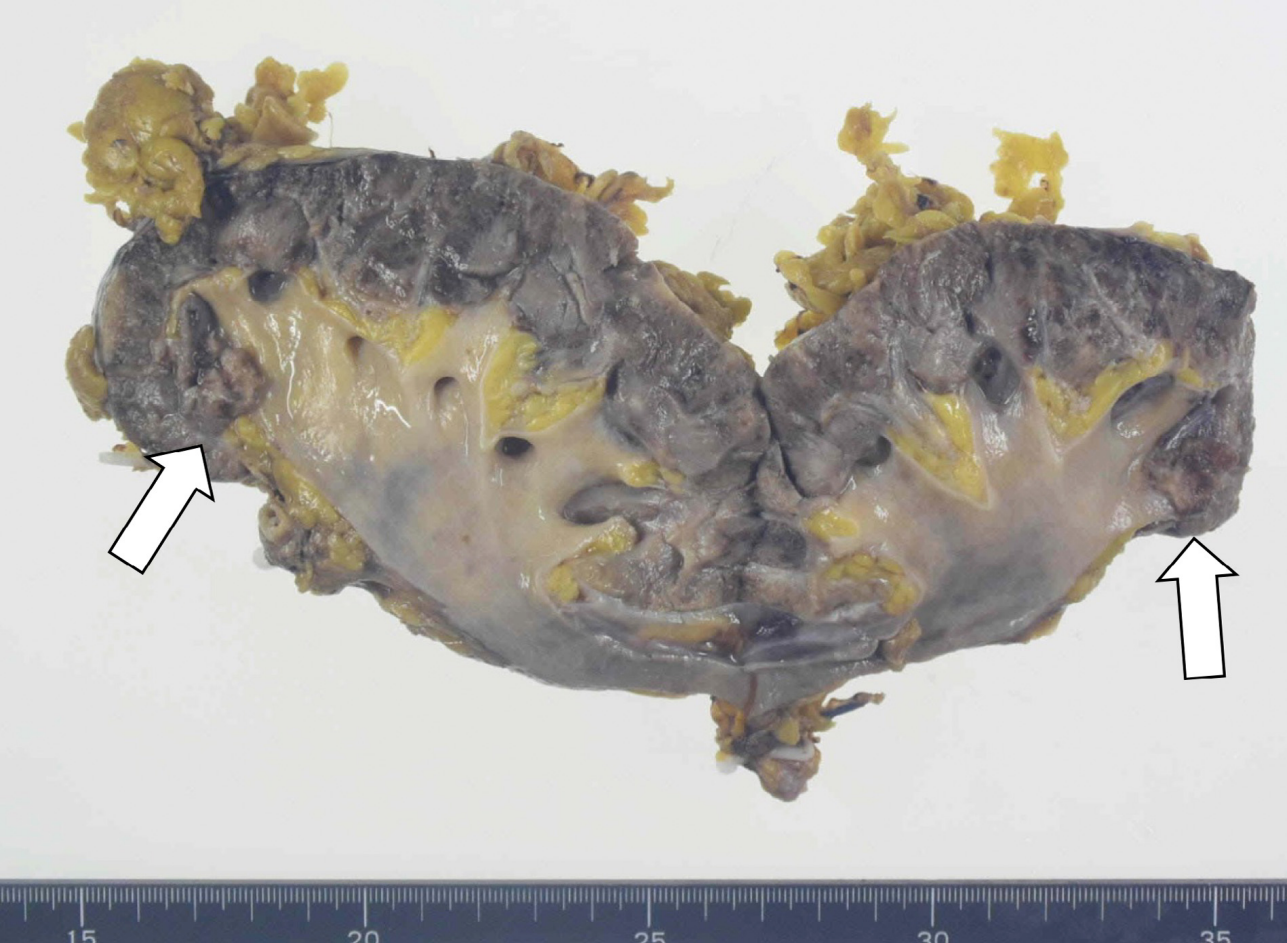
Specimen of the tumors. White arrows show the renal pelvis tumors.

An 88‐year‐old woman with gross hematuria was diagnosed with a renal pelvis tumor by contrast‐enhanced CT at another hospital (Fig. 1). Intermittent gross hematuria and back pain associated with hematuria, which was treated symptomatically, including blood transfusions. She was transferred and admitted to our hospital 6 months after the diagnosis of the renal pelvis tumor because of family requests. Her ECOG PS was 2. She was not taking any medications, including anticoagulants. Her Hb was 6.7 g/dL revealing severely anemia, resulting in heart failure. Blood transfusion for anemia due to persistent hematuria was done. On cystoscopy, a bladder tumor was not detected, but mild hematuria from a right orifice was detected. Therefore, hematuria caused by the renal pelvic tumor. The staff anesthesiologist confirmed that she was tolerant to laparoscopic nephrectomy with general anesthesia. The patient and her family requested to undergo surgical treatment, not radiation. Therefore, we planned to perform a laparoscopic nephrectomy 2 weeks after admission. However, 1 week after admission, she developed a fever and right back pain with disappearance of hematuria. The vital signs showed tachycardia (123 bpm), tachypnea (23 rpm), and fever (40.2°C). Her WBCs were 10 600/μL, Hb was 7.4 g/dL, and platelet decreased from 170 000/μL on the previous day to 110 000/μL. Serum creatinine level increased from 0.67 mg/dL on the previous day to 0.94 mg/dL. Her score of Japanese Association for Acute Medicine's acute stage DIC diagnostic criteria was 3. CT showed blood clots in the renal pelvis and ureter (Fig. 2). The absence of hematuria on manual bladder irrigation suggested that the blood filling the renal pelvis and ureter was clotted. She was diagnosed with pre‐DIC due to acute obstructive pyelonephritis that might not be sufficiently drained by a ureteral stent. We decided to perform an emergency right nephrectomy to control infection due to the family's desire for a reliable treatment. The patient was predicted to bleed easily due to abnormal coagulation caused by the infection. We selected laparoscopic nephrectomy to reduce intraoperative bleeding due to pneumoperitoneal pressure. We also planned to convert to hand‐assisted laparoscopic surgery or open surgery when the dissection did not progress intraoperatively. Retroperitoneoscopic nephrectomy using 4‐ports was performed in the lateral position. Intraoperatively, the perirenal area was easy to bleeding as predicted preoperatively (Fig. 3a). There was moderate adhesion around the renal artery. The right renal artery was dissected with repeated blunt dissection of tissues around the renal artery, and Hem‐o‐lok clips were clipped on the renal artery. Subsequently, the amount of bleeding from the perirenal area decreased (Fig. 3b). The ureter was dissected as caudally as possible, and the diseased kidney and the ureter removed. Total operating time was 192 min and estimated blood loss was 525 mL. The interior of renal pelvis and ureter were filled with blood clots. Papillary tumors of 16 × 13 mm and 13 × 11 mm were detected in the upper calyx of the right kidney (Fig. 4). Intraoperatively, 4 units of mannitol adenine phosphate, 4 units of fresh frozen plasma, and 10 units of platelet transfusion were performed. The patient was recovered from fever the day after surgery and discharged on postoperative day 15 without major complications. *Escherichia coli* was detected in the blood culture before surgery. Pathologic diagnosis was urothelial carcinoma, pTa, low grade. Significant chronic and acute inflammation were observed in the renal pelvis, renal parenchyma, and perirenal fat.

## Discussion

In the present case, the patient had bacteremia due to acute obstructive pyelonephritis caused by blood clots. Although drainage with a ureteral stent or nephrostomy is a common treatment for obstructive pyelonephritis, drainage of the obstruction caused by blood clots filling the renal pelvis and ureter might not be effective. Therefore, emergency nephrectomy was performed. To the best of our knowledge, this is the first report of an emergency laparoscopic nephrectomy for a patient with acute obstructive pyelonephritis with bacteremia due to bleeding from a renal pelvic cancer.

Kamei *et al*. reported that analyses of a national database revealed a 2‐day delay in drainage of obstructive pyelonephritis increases the fatality rate.^2^ It has also been reported that obstructive pyelonephritis is more severe in patients over 85 years of age.^3^ The patient was 88 years old and had coagulopathy due to acute obstructive pyelonephritis that could not be drained, so immediate nephrectomy was required. Laparoscopic nephrectomy for an infected kidney has been reported.^4^, ^5^ Manohar *et al*. reported that laparoscopic nephrectomy via transperitoneal approach for an infected kidney with enlarged hilar lymph nodes resulted in a higher rate of open conversion and more blood loss compared to patients without such an enlarged hilar lymph node.^4^ They speculate that the enlarged hilar lymph nodes are causing displacement of the hilar structures.^4^ Since there was no history of obstructive pyelonephritis in this patient, we considered that the perirenal adhesions were mild. The perirenal adhesions around the renal arteries on CT images were not considered to be severe (Fig. 2a). No enlarged lymph nodes in the renal hilum were observed on retrospective view. Intraoperatively, there was moderate adhesion around the renal artery, but no displacement of structures in the renal hilum. In addition, the patient had a sudden coagulopathy due to acute obstructive pyelonephritis, which would have resulted in massive bleeding if nephrectomy were performed by laparotomy. Therefore, we selected laparoscopic surgery. If the laparoscopic approach failed to dissect the nephrectomy due to adhesions or bleeding, we planned to sift to hand‐assisted laparoscopic approach. Hand‐assisted laparoscopic surgery has been reported to be effective even in patients with strong perirenal adhesions such as nephrocutaneous fistula.^6^ Intraoperatively, the patient had moderate adhesions around the renal artery and was easy to bleeding due to abnormal coagulation. However, the bleeding from the perirenal area diminished after the artery was clipped. Laparoscopic nephrectomy is an option for the acute nephrectomy in the setting of undrainable obstructive pyelonephritis with coagulopathy.

## Conclusion

Renal pelvis carcinomas with hemorrhage requiring blood transfusion are at risk of developing acute obstructive pyelonephritis. Therefore, renal pelvis carcinomas with hemorrhage should be treated with radical nephroureterectomy as early as possible.

## Author contributions

Masato Yanagi: Conceptualization; data curation; writing – original draft. Ken Takahashi: Conceptualization; data curation. Yuta Kikuchi: Data curation. Takuya Takahashi: Data curation. Hideaki Iwata: Data curation. Hiro Izuta: Data curation. Shuichi Osawa: Conceptualization; data curation. Taiji Nishimura: Writing – original draft. Yukihiro Kondo: Writing – original draft; writing – review and editing.

## Conflict of interest

The authors declare that they have no competing interests.

## Approval of the research protocol by an Institutional Reviewer Board

Not applicable.

## Informed consent

Informed consent for publication has been obtained from the patient.

## Registry and the Registration No. of the study/trial

Not applicable.
